# Toxic chemical weapons of assassination and warfare: nerve agents VX and sarin

**DOI:** 10.1080/24734306.2017.1373503

**Published:** 2017-09-07

**Authors:** Peter R. Chai, Edward W. Boyer, Houssam Al-Nahhas, Timothy B. Erickson

**Affiliations:** a Department of Emergency Medicine, Division of Medical Toxicology, Brigham & Women’s Hospital, Harvard Medical School, Boston, MA, U.S.A; b UOSSM International, Union of Medical Care and Relief Organizations, Syria; c Cerrahpasa Medical School, Istanbul University, Turkey; d Harvard Humanitarian Initiative, Harvard University, Cambridge, MA, U.S.A

**Keywords:** Nerve agents, sarin, VX, antidotes

## Abstract

The use of VX and sarin as weapons of assassination and warfare raises important considerations for healthcare professionals who may encounter victims, bystanders, and responders who require prompt assessment and treatment. Chemical warfare agents such as VX and sarin constitute a considerable threat to the health of the civilian population, military personnel, and peacekeeping forces. Healthcare providers should recognize symptoms of nerve agent exposure, understand regional and international notification procedures for potential attacks, as well as the indications for and available supply of antidotal therapy.

In February 2017, Kim Jong-Nam, the half-brother of North Korea’s ruler Kim Jong-Un, was allegedly assassinated inside the Kuala Lumpur airport. Two women coated their hands with a mysterious chemical and wiped Nam’s face as he was transiting through the airport. Nam presented for emergency care at the airport and quickly decompensated and died. An autopsy identified the nerve agent ethyl *N*-2-diisopropylaminoethyl methylphosphonothiolate (VX) in ocular and facial swabs in the decedent [1]. Kim Jong-Nam’s dramatically fatal poisoning was not the first intentional poisoning with VX. The *Aum Shinrikyo* cult used VX to kill dissenting members in 1994 and 1995. Later in 1995, *Aum Shinrikyo* released sarin (O-isopropyl methylphosphonofluoridate), a nerve agent similar to VX, in the Tokyo subway system resulting in 640 people taken to the nearest hospital for evaluation and treatment [2]. In 2013, and most recently in April 2017, the Syrian government attacked civilians, including young children, with sarin [3]. These chemical attacks resulted in large groups of exposed individuals presenting for emergency care that overwhelmed the capacity of hospitals and depleted antidote stock. The use of VX and sarin as weapons of assassination and warfare raises important considerations for healthcare professionals who may encounter victims, bystanders, and responders who require prompt assessment and treatment. Here, we review the basic pathophysiology of nerve agents, discuss antidotal therapy, and cover adjunct treatments that can be used in the event an antidote stock is depleted.

VX and sarin are odorless organophosphates developed as chemical warfare nerve agents. VX is a “binary” agent that requires mixing of stable (and nontoxic) precursors immediately prior to use to produce the toxic agent. Because each assassin wiped Kim Jong-Nam’s face and survived, it is likely that each attacker carried one of the precursors that reacted on the victim’s face to produce VX. VX has low volatility (long environmental persistence) [4], while sarin is highly volatile (easily aerosolized) and therefore less stable in the environment. Compared to sarin, the V–type of organophosphorus nerve agents (V standing for venomous) are more lethal.

Dermal, ocular, and inhalational exposure can lead to organophosphate toxicity. The lethal dose (LD50) for VX ranges from as little as 10 mg in dermal exposures to 25–30 mg if inhaled. Organophosphates inhibit acetylcholinesterase by covalently binding to the enzyme’s active site. Accumulation of acetylcholine activates cholinergic synapses to produce a cholinergic toxidrome (Figure 1). Death from VX and sarin occurs through respiratory arrest or neurotoxicity. Persistent binding of VX and sarin to the active site irreversibly inhibits acetylcholinesterase, a process known as “aging.” Symptoms appear within a few seconds after exposure to vapor forms of VX or sarin, and within a few minutes to hours after exposure to liquid VX. Any nerve agent contact to the skin, unless washed off immediately, could be lethal. Video footage from social media during the Syrian chemical attacks shows classic signs of a cholinergic toxidrome: diaphoresis, convulsions, bronchorrhea, and bradycardia. As demonstrated in the Syrian conflict, children are more susceptible than adults because of smaller body mass, higher respiratory rate, increased skin permeability, and immature metabolic systems [5].

Given the difficulty in manufacturing and storing nerve agents, potential exposures to VX or sarin typically occur in the context of chemical warfare. Even an assassination attempt as brazen as that of Kim Jong Nam is concerning given the long persistence of VX in the environment; innocent bystanders can become inadvertently exposed and may develop symptoms. Any exposure to VX or sarin therefore, should be considered potentially lethal, and aggressive supportive measures and antidotes need to be immediately administered. First responders should ensure correct personal protective equipment prior to approaching the scene of a potential nerve agent exposure. Because dermal exposure carries the greatest risk, exposed individuals should be promptly removed from the suspected source and aggressively decontaminated using soap and water. Exposed clothing should be removed and secured in a sealed biological plastic bag to prevent re-exposure.

Medical management of nerve-agent poisoned casualties is derived from clinical experience with organophosphate pesticide poisoning [6]. The two pillars of treatment include parenteral administration of atropine (2–6 mg every 5–10 minutes) to counter the muscarinic effects of excess acetylcholine, and 1–2 g of pralidoxime (2-PAM) to cleave VX and sarin from acetylcholinesterase, restore the active site, and prevent aging. Atropine should be administered until symptoms of bradycardia, bronchospasm, and bronchorrhea resolve, a process that may require extraordinarily high doses of atropine. Due to a permanently charged pyridinium motif, 2-PAM can neither cross the blood– brain barrier nor restore cholinesterase activity in the brain. Investigators have recently discovered novel oximes that penetrate the blood–brain barrier and provide 24-hour survival superior to 2-PAM when challenged with lethal dosages of the sarin and VX surrogates [7].

A flood of victims after a VX or sarin exposure may quickly deplete hospital supplies of atropine and 2-PAM. Healthcare providers should be aware that, if necessary, these antidotes may be used emergently beyond their labeled expiration dates [8]. Under the US Food and Drug Administration (FDA) Shelf Life Extension Program, only those antidotes kept in “push packs” under contracted conditions of temperature and humidity control have official extension of their shelf lives, but likely the stability of these xenobiotics if stored under standard pharmacy conditions will last for many years after the expiration date. Alternative sources for atropine may include veterinary centers. Treatment with anticholinergic drugs such as diphenhydramine that penetrate the blood–brain barrier may offer benefit. Diazepam may treat organophosphate-induced respiratory depression and seizures and increases survival in animal models. VX and sarin are highly lipid soluble; this physicochemical property may allow intravenous lipid emulsion to sequester and potentially blunt toxicity [9].

Chemical warfare agents such as VX and sarin constitute a considerable threat to the health of the civilian population, military personnel, and peacekeeping forces. Healthcare providers should recognize symptoms of nerve agent exposure, understand regional and international notification procedures for potential attacks, and the indications for antidotal therapy.

## Figures and Tables

**Figure 1:**
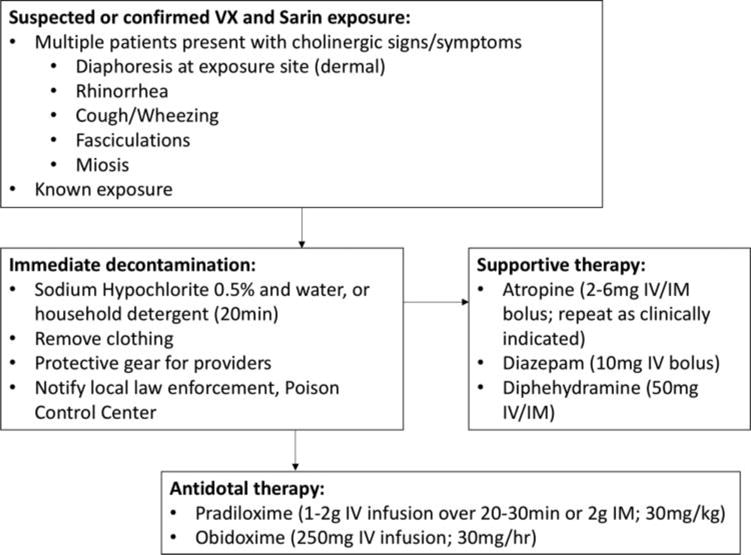
Clinical findings and therapy for nerve agents VX and sarin.
